# Analysis of Polarization Detector Performance Parameters on Polarization 3D Imaging Accuracy

**DOI:** 10.3390/s23115129

**Published:** 2023-05-27

**Authors:** Pengzhang Dai, Dong Yao, Tianxiang Ma, Honghai Shen, Weiguo Wang, Qingyu Wang

**Affiliations:** 1 Key Laboratory of Airborne Optical Imaging and Measurement, Chinese Academy of Sciences, Changchun 130033, China; daipengzhang19@mails.ucas.ac.cn (P.D.);; 2 Changchun Institute of Optics, Fine Mechanics and Physics, Chinese Academy of Sciences, Changchun 130033, China; 3 University of Chinese Academy of Sciences, Beijing 100039, China

**Keywords:** polarization, 3D reconstruction accuracy, normal vector, diffuse reflection, detector performance parameters

## Abstract

Three-dimensional (3D) reconstruction of objects using the polarization properties of diffuse light on the object surface has become a crucial technique. Due to the unique mapping relation between the degree of polarization of diffuse light and the zenith angle of the surface normal vector, polarization 3D reconstruction based on diffuse reflection theoretically has high accuracy. However, in practice, the accuracy of polarization 3D reconstruction is limited by the performance parameters of the polarization detector. Improper selection of performance parameters can result in large errors in the normal vector. In this paper, the mathematical models that relate the polarization 3D reconstruction errors to the detector performance parameters including polarizer extinction ratio, polarizer installation error, full well capacity and analog-to-digital (A2D) bit depth are established. At the same time, polarization detector parameters suitable for polarization 3D reconstruction are provided by the simulation. The performance parameters we recommend include an extinction ratio ≥ 200, an installation error ∈ [−1°, 1°], a full-well capacity ≥ 100 $Ke^{-}$, and an A2D bit depth ≥ 12 bits. The models provided in this paper are of great significance for improving the accuracy of polarization 3D reconstruction.

## 1. Introduction

With the rapid development of photoelectric imaging technology, two-dimensional (2D) images represented by light intensity information cannot satisfy the user’s desire to explore the real world [1]. Therefore, many three-dimensional (3D) imaging technologies have been created. Generally speaking, 3D imaging technology can be divided into active and passive 3D imaging. Active 3D imaging technology uses an active light source to collect light-field images for 3D reconstruction while passive 3D-imaging uses natural light to collect light-field images. Typical active 3D-reconstruction methods include laser radar 3D reconstruction [2,3,4] and structured-light 3D reconstruction [5,6] and so on. However, the reconstruction accuracy of these two methods is inversely proportional to the distance and their application cost is high [7]. Typical passive 3D-imaging includes stereo vision [8] and shape from shading [9,10], but these two methods have low reconstruction accuracy and extremely depend on the texture details of the object.

With the understanding of light polarization, researchers have found that the normal vector of an object can be calculated using the polarization property of light. Polarization imaging has strong light weakening and weak light strengthening characteristics, so accurate polarization information can be obtained even if the external light source information is not ideal. Meanwhile, 3D reconstruction based on polarization does not depend on the texture characteristics of the object and can achieve good results for an object with low texture and high reflection.

In the initial stage, polarization 3D imaging technology mainly used the polarization characteristics of specular reflection. In 1979, Koshikawa [11] established the mapping relationship between the degree of polarization of reflected light and the surface vector of an object. Then, Wolff [12,13] used Fresnel’s theory to establish a mathematical model involving the reflected light of the object surface and its normal vector. Since this model was established, many research teams have conducted 3D reconstruction using metal, glass, and additional material-type objects with a specular reflection [14,15,16]. However, the mapping relation between the polarization degree and zenith angle based on the specular reflection model faces multiple value ambiguity. Moreover, experiments require setting up a complex light source.

In 1995, Partridge [17] solved the ambiguity problem of the zenith angle by using diffuse light transmitted from the interior of an object. In 2006, Atkinson [18,19] used the polarization characteristics of diffused light to realize 3D reconstruction. The mapping relation between the polarization degree of diffuse reflection and the zenith angle was derived. However, the method still shows ambiguity when calculating the azimuth angle. Studies show [20] that it is impossible to calculate the 3D shape by only relying on polarization information, and the disambiguation process needs to be completed by other means, including the photometric stereo method [21,22], and shadow reconstruction method [10,23] etc. Photometric stereo is a method restoring the surface of an object using images with different photometric information under different light sources and is commonly used as a 3D reconstruction method. Shape from shading is a method based on weak structured light. This method captures the moving shadow by moving the object in front of the light source, and then observes the spatial position of the shadow, to reconstruct the 3D structure of the object. A series of subsequent studies based on these methods have been published and have been the focus of polarization 3D imaging for the last decade. Typical work, such as that by Atkinson [19], proposed combining photometric stereoscopic vision with diffuse polarization information for surface shape reconstruction. Mahmoud [24] used shading information to assist diffuse light polarization information for 3D reconstruction. Smith [25] proposed a surface depth estimation method based on sparse linear formulas to calculate the azimuth angle ambiguity problem. Kadambi [26] used the Kinect depth camera to correct the azimuth angle. In the last three years, some scholars have used polarized images as input to calculate the normal vector using the powerful feature extraction capabilities of deep learning technology. Representative works of this type can be found in Refs. [27,28,29]. However, due to the difficulty in creating the datasets required for such work, current public datasets are small in size and not ideal for practical use.

At present, the main idea of the polarization 3D reconstruction method is still to use the polarization information of diffuse light. In theory, polarization 3D imaging can achieve high precision, but in practice, due to the limitation of the performance of polarization detectors, the actual results are often not ideal. According to the Ref [27]., the optimal results that various algorithms can achieve at present are shown in Table 1. Even if we use deep learning methods, the normal vector error is still very high.

Strictly speaking, polarization 3D imaging based on diffuse reflection requires very high precision of the polarization detector. We will explain it in Section 2.2. Not all polarization detectors can complete the task of polarization 3D imaging. Many scholars have found that the performance parameters of the detector would affect the quality of polarization 3D imaging [20,31,32], but when doing experiments, they only select the corresponding parameters based on experience. Through the above-mentioned references and the authors’ experiments, it was found that the polarizer extinction ratio, the polarizer Installation error, the full-well capacity, and the A2D bit depth considerably impact the 3D reconstruction accuracy. This also explains why the experimental results of reference [20,24,25,30,31] are so different from the true value.

Based on the above considerations, this paper decides to quantitatively analyze the relationship between polarization detector performance parameters and polarization 3D imaging performance. Although many researchers have studied the accuracy of the Stokes vector or degree of polarization [33,34,35,36,37,38,39], only studying the accuracy of Stokes vectors or degree of polarization is of limited help to polarization 3D imaging. We must explore the quantitative relationship between the performance parameters of polarization detectors and the accuracy of polarization 3D imaging. However, to the best of the authors’ knowledge, there is currently no published work providing a quantitative relationship that describes the impact of polarization detector performance parameters on 3D-polarization imaging accuracy. Obviously, such a quantitative relationship is very important in the field of polarization 3D imaging.

Based on the above considerations, in this paper, we focus on establishing mathematical models of the relationship between polarization 3D imaging accuracy and performance parameters of the polarization detector including extinction ratio, polarizer installation error, full-well capacity, and A2D bit depth. In addition, we simulate and analyze the surface normal vector error under various parameters and introduce the following polarization detector parameters suitable for polarization 3D reconstruction: an extinction ratio ≥ 200, an installation error ≤ 1°, a full-well capacity ≥ 100 $Ke^{-}$, and an A2D bit depth ≥ 12 bits. Meanwhile, we prove that the actual (experimental) error is consistent with the theoretical error given in this paper. The error models proposed in this paper provide important theoretical support for the improvement of the accuracy of polarization 3D imaging, especially for single imaging represented by airborne polarization remote sensing 3D imaging. At the same time, in the era of artificial intelligence, the models of this paper also provide theoretical guidance for the production of the next generation polarization 3D imaging dataset.

Our contributions are summarized as follows. This is the first attempt to systematically analyze the error sources of polarization 3D imaging and to establish a mathematical model of the relationship between polarization 3D imaging accuracy and polarization detector parameters (Section 3). The error of polarization 3D imaging under various parameters is simulated and analyzed (Section 4). The accuracy of the error model is evaluated by experiments (Section 5).

## 2. Preliminary Knowledge

In this section, we first introduce the basic theory of polarization and polarization 3D imaging based on diffuse light and some other basic knowledge related to this paper.

### 2.1. Representation of Polarization

The Stokes vector is used to describe the polarization of light, which is defined as follows [40]:

(1) $$\vec{S}=\left[\begin{array}{c} S_{0}\\ S_{1}\\ S_{2}\\ S_{3} \end{array}\right]=\left[\begin{array}{c} I_{0}+I_{90}\\ I_{0}-I_{90}\\ I_{45}-I_{135}\\ I_{RHC}-I_{LHC} \end{array}\right],$$

where *I*_0_, *I*_45_, *I*_90_, and *I*_135_ are the radiances measured through polarizers oriented at 0, 45, 90, and 135°, respectively.

The degree of linear polarization (DoLP) and the angle of linear polarization (AoLP) are defined as:

(2) $$DoLP=\frac{\sqrt{S_{1}^{2}+S_{2}^{2}}}{S_{0}}.$$

(3) $$\;AoLP=\frac{1}{2}arctan(\frac{S_{2}}{S_{1}}).$$

For the convenience of subsequent derivation, we have adopted a unified symbol system: ρ = DoLP, and φ = AoLP.

### 2.2. Object Surface Normal Vector

**Figure 1:**
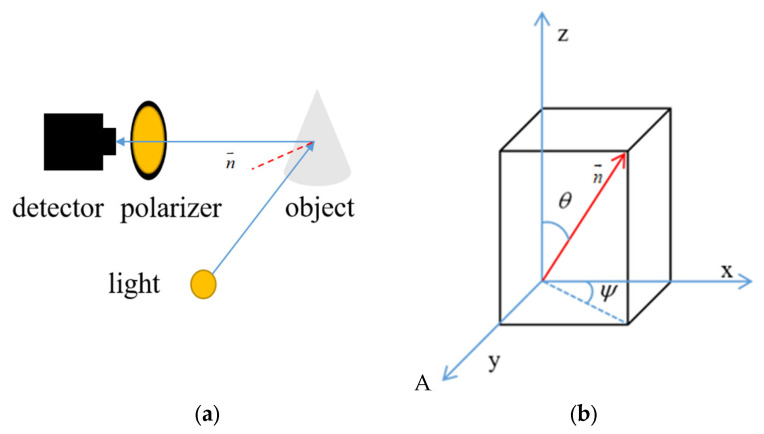
Object surface normal vector. (**a**) surface normal vector diagram (**b**) normal vector in the coordinate system.

The basic consensus of 3D reconstruction is that the 3D morphology of an object surface can be recovered by the normal vector of the object surface [41]. Meanwhile, the normal vector can be described by two angles in the spatial coordinate system: the zenith angle (θ) and the azimuth angle (ψ). Figure 1 shows a schematic indicating the normal vector. The coordinate system is the imaging plane coordinate system, wherein a normal vector is expressed as:

(4) $$\vec{n}=\;\left[\begin{array}{c} \sin\theta \cos\psi \\ \sin\theta \sin\psi \\ \cos\theta \end{array}\right].$$

### 2.3. Basic Principles of Diffuse Polarization 3D Imaging

According to the study of diffuse polarization 3D imaging by [18,19,20,25,32], under the condition of non-polarized light source, the mapping between the DoLP of diffuse light and the zenith angle is:

(5) $$\rho =\frac{{\left(\eta -\frac{1}{\eta }\right)}^{2}\sin^{2}\theta }{2+2\eta^{2}-{\left(\eta +\frac{1}{\eta }\right)}^{2}\sin^{2}\theta +4\cos\theta \sqrt{\eta^{2}-\sin^{2}\theta }},$$

where η is the index of refraction, and θ is the zenith angle. The Inverse function is [20]:

(6) $$\theta (\rho ,n)=\mathrm{arc}\cos\left(\sqrt{\frac{1+2\rho +\rho^{2}-2\eta^{2}+2\eta^{2}\rho +4\eta^{2}\rho^{2}-4\eta^{3}\rho \sqrt{1-\rho^{2}}+\eta^{4}-\eta^{4}\rho^{2}}{1+2\rho +\rho^{2}-2\eta^{2}+4\eta^{2}\rho +6\eta^{2}\rho^{2}+\eta^{4}+2\eta^{4}\rho +\eta^{4}\rho^{2}}}\right).$$

Equation (6) is used to calculate the zenith angle through ρ. For non-conductive material, η is generally between 1.4–1.6, and η = 1.5 is generally used in practical applications.

The mapping between the φ of diffuse light and the azimuth angle is given below [20]:

(7) $$\psi =\left\{\begin{array}{l} \varphi \;\;if\;\;-\frac{\pi }{2}\leq \psi <\frac{\pi }{2}\;\\ \varphi -\pi \;\;if\;\;\frac{\pi }{2}\leq \psi <\frac{3\pi }{2}\\ \varphi +\pi \;\;if\;\;-\frac{3\pi }{2}\leq \psi <-\frac{\pi }{2}\; \end{array}\right.,$$

where *ψ* is the azimuth angle. Equations (5) and (7) are the basic equations for calculating the normal vector using diffuse polarization. 

**Figure 2:**
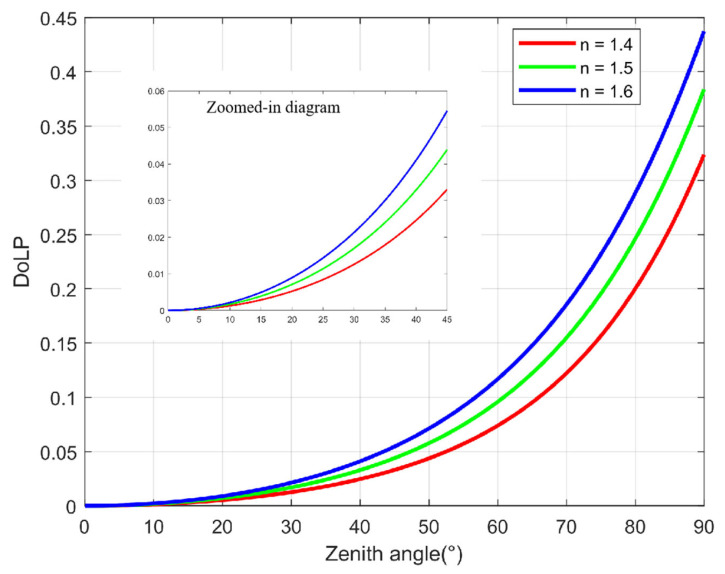
Mapping between the ρ and zenith angle.

The function graph of Equation (5) is shown in Figure 2. It can be seen from Figure 2 that the smaller the ρ, the easier the zenith angle will be disturbed by the ρ error. Table 2 clearly shows this conclusion. We assume that the error of ρ is equal to 0.005. When the ρ equals to 0.1, the zenith angle error is 1.0445°, and when the ρ equals to 0.01, the zenith angle error will be 6.6150°. Usually, such interference is unavoidable due to the influence of polarization detector performance parameters such as the polarizer extinction ratio (ER), polarizer installation error, full well size capacity, and A2D bit depth.

### 2.4. Fundamental of Error Propagation

The principle of error propagation is a commonly used mathematical tool in engineering measurement, and its mathematical expression is [42]:

(8) $${\left.\sigma_{f}=\sqrt{{\left(\frac{\partial f}{\partial x_{1}}\right)}^{2}\sigma_{x_{1}}^{2}+{\left(\frac{\partial f}{\partial x_{2}}\right)}^{2}\sigma_{x_{2}}^{2}+{\left(\frac{\partial f}{\partial x_{3}}\right)}^{2}\sigma_{x_{3}}^{2}+\sum_{1\leq i<j\leq 3}\rho_{ij}\sigma_{x_{i}}\sigma_{x_{j}}}\right|}_{x_{i}=\bar{x_{i}}},$$

where f is the dependent variable, $x_{i}$ is the independent variable, $\rho_{ij}$ is the dependency coefficient, $\sigma_{f}$ is the standard deviation of f, and $\sigma_{xi}$ is the standard deviation of xi. When the measured variables are independent random variables, $\rho_{ij}$ is equal to 0. Error propagation equation is strictly accurate for normal distributed random variables. However, with the increase in the number of random events in unit time, the Poisson distribution will gradually approximate to the normal distribution in which the mean and variance are equal to λ.

## 3. Influence of Polarization Detector Performance Parameters on Polarization 3D Reconstruction

According to Section 2, even a small error at a small ρ will cause a large error in the normal vector. In an actual polarization detection process, these errors are unavoidable, and the error magnitude is closely related to the polarization detector performance parameters. In order to explore the influence of these performance parameters on polarization 3D imaging accuracy, the mathematical models between the performance parameters and error in zenith and azimuth angles are established in this section.

### 3.1. Influence of Polarizer Extinction Ratio on Polarization 3D Reconstruction Accuracy

According to [37], the extinction ratio is an important factor affecting polarization imaging quality. Although [37] discussed the influence of extinction ratio on polarization imaging accuracy, there is no quantitative model that describes the relationship between the extinction ratio and polarization 3D imaging accuracy. This section will quantitatively describe the relationship between the extinction ratio and polarization 3D imaging accuracy.

According to Malus’ law [40], the light intensity accepted by an ideal polarization pixel is defined as:

(9) $$I_{\alpha }=\frac{1}{2}S_{0}^{in}[1+\rho^{in}\cos2(\alpha -\varphi )]\;,$$

where α is the angle between the polarizer pass axis and the horizontal reference axis of the system (α equals to 0, 45, 90, and 135°) and $S_{0}^{in}$, *φ*, and $\rho^{in}$ are the intensity, AoLP, and DoLP of the incident light, respectively.

Due to current technological limitations, there is no perfect polarizer. In theory, there are two parameters used to describe the linear polarization ability of a polarizer: the extinction ratio (*ER*) and diattenuation (*D*). These parameters can be described by the following mathematical formula:

(10) $$ER=\frac{q}{r},$$

(11) $$D=\frac{q-r}{q+r}=\frac{ER-1}{ER+1},$$

where *q* is the major transmittance and *r* is the minor transmittance. In the ideal case, *q* = 1 and *r* = 0. When the ideal condition is not satisfied, the expression of the light intensity detected by the detector becomes:

(12) $$I_{\alpha }=\frac{1}{2}S_{0}^{in}[(q+r)+(q-r)\rho^{in}\cos2(\alpha -\varphi )].$$

For α = 0, 45, 90, and 135° in rotating polaroid measurement system, the light intensity detected by the polarization detector is:

(13) $$\begin{array}{l} I_{0}=\frac{1}{2}S_{0}^{in}[(q+r)+(q-r)\rho^{in}\cos2(\varphi )]\\ I_{45}=\frac{1}{2}S_{0}^{in}[(q+r)+(q-r)\rho^{in}\sin2(\varphi )]\\ I_{90}=\frac{1}{2}S_{0}^{in}[(q+r)-(q-r)\rho^{in}\cos2(\varphi )]\\ I_{135}=\frac{1}{2}S_{0}^{in}[(q+r)-(q-r)\rho^{in}\sin2(\varphi )] \end{array},$$

respectively. In Equation (13), we assume that the values of q and r are equal in the four directions, which is correct in the rotating polaroid imaging system, but obviously not completely correct in the Division of Focal Plane (DoFP) detectors. However, in a large pixel group, the differences of the q and r in four pixels are relatively small. Take the Sony IMX 250 mzr [43] polarization detector as an example, in a large pixel group, the difference of q value in four directions is less than 0.0010 and the difference of r in four directions is less than 0.0015. Based on the above examples, Equation (13) also has certain applicability in DoFP.

Substituting Equation (13) into Equation (1), we get the Stokes vector of the incident light:

(14) $$\begin{array}{l} S_{0}=\frac{1}{2}S_{0}^{in}[2(q+r)]\\ S_{1}=\frac{1}{2}S_{0}^{in}[2(q-r)\rho^{in}\cos2(\varphi )]\\ S_{2}=\frac{1}{2}S_{0}^{in}[2(q-r)\rho^{in}\sin2(\varphi )] \end{array}.$$

Substitute Equation (14) into Equations (2) and (3), we could get the detected ρ and φ:

(15) $$\rho^{{}_{{}^{\mathrm{detected}}}}=\frac{ER-1}{ER+1}\rho^{in},$$

(16) $$\varphi^{\mathrm{detected}}=\varphi^{in},$$

where $\rho^{{}_{{}^{\mathrm{detected}}}}$ is the ρ detected by the polarization detector. In the same way, $\varphi^{\mathrm{detected}}$ is the φ detected by the polarization detector.

According to Equations (15) and (16), the extinction ratio of the polarizer only affects the ρ and has no influence on the φ. Therefore, it can be concluded that the extinction ratio of polarization detectors affects the accuracy of the zenith angle but has no influence on the azimuth angle.

For a linear polarizer with an extinction ratio of ER, the zenith angle error is:

(17) $$\theta_{error}^{ER}=\theta (\rho^{\mathrm{detected}})-\theta (\rho^{in}).$$

where $\theta_{error}^{ER}$ is the zenith error caused by ER and θ(ρ,n) is Equation (6). What we need to pay attention to is that ER is not a random value, and the error of ρ caused by ER can be corrected by Equation (15). However, we need to take into account the following points: (a) For the DoFP polarization camera, we need to measure ER with the help of an integrating sphere, which is troublesome in engineering; (b) For DoFP detectors, each large pixel group has an ER, so we need to correct ER point by point during programming, which increases the computational complexity. (c). The actual corrected results are often not particularly ideal. After considering these factors, we decided to choose an appropriate extinction ratio to replace the method of point-by-point correction. Of course, which method to choose is determined by the users. The importance of this section is only to guide the users to choose the appropriate extinction ratio after abandoning the idea of correction through Equation (15).

### 3.2. Influence of Polarizer Installation Error on Polarization 3D Reconstruction Accuracy

**Figure 3:**
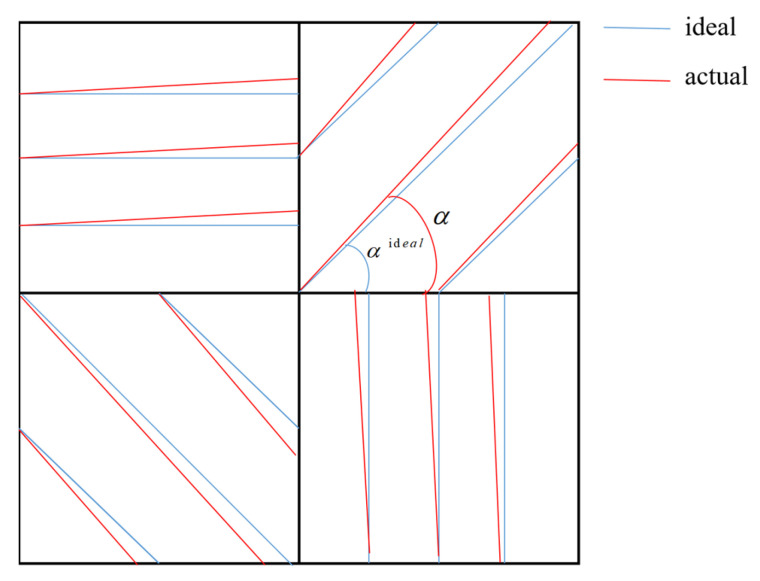
Polarizer direction error of the DoFP detector.

**Figure 4:**
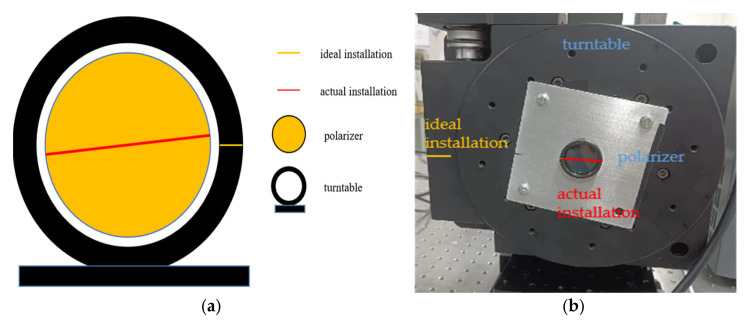
Polarizer installation error for rotating polarizer imaging system; (**a**) diagram illustrating the installation error; (**b**) photograph of actual installation error.

The installation errors of polarizers are unavoidable, and we can refer to Figure 3 and Figure 4 to understand such errors. We define the installation error of a polarizer as:

(18) $$\;∆\alpha =\alpha -\alpha^{\mathrm{ideal}}.$$

where $\alpha^{ideal}$ is the ideal installation angle and α is the actual installation angle.

In order to analyze the effect of installation error alone, we ignore the effect of ER. We assume q=1,r=0. This assumption is reasonable, because from the simulation result in Section 4.1, we will find that when the ER is greater than 300, the influence of ER on the zenith angle is very weak. Taking the common commercial polarization detector Sony IMX250mzr as an example, in the visible light band, the extinction ratio of its central wavelength is greater than 300.

Then, the light intensity obtained by the polarization detector becomes:

(19) $$\begin{array}{l} I_{0}=\frac{1}{2}S_{0}^{in}[1+\rho^{in}\cos2(\varphi +∆\alpha_{0})]\\ I_{45}=\frac{1}{2}S_{0}^{in}[1+\rho^{in}\sin2(\varphi +∆\alpha_{45})]\\ I_{90}=\frac{1}{2}S_{0}^{in}[1-\rho^{in}\cos2(\varphi +∆\alpha_{90})]\\ I_{135}=\frac{1}{2}S_{0}^{in}[1-\rho^{in}\sin2(\varphi +∆\alpha_{135})] \end{array}.$$

The Stokes vector becomes:

(20) $$\begin{array}{l} S_{0}=\frac{1}{2}S_{0}^{in}\{2+\rho^{in}[\cos2(\varphi +∆\alpha^{0})-\cos2(\varphi +∆\alpha^{90})]\}\\ S_{1}=\frac{1}{2}S_{0}^{in}\{\rho^{in}[\cos2(\varphi +∆\alpha^{0})+\cos2(\varphi +∆\alpha^{90})]\}\\ S_{2}=\frac{1}{2}S_{0}^{in}\{\rho^{in}[\sin2(\varphi +∆\alpha^{45})+\sin2(\varphi +∆\alpha^{135})]\} \end{array}.$$

By substituting Equation (20) into Equation (2), we can get the detected ρ:

(21) $$\rho^{\mathrm{detected}}=\frac{\rho^{in}\sqrt{{\left[\cos2(\varphi +∆\alpha_{0})+\cos2(\varphi +∆\alpha_{90})\right]}^{2}+{\left[\sin2(\varphi +∆\alpha_{45})+\sin2(\varphi +∆\alpha_{135})\right]}^{2}}}{\left\{2+\rho^{in}[\cos2(\varphi +∆\alpha_{0})-\cos2(\varphi +∆\alpha_{90})\right]}.$$

The zenith angle error is calculated as:

(22) $$\;\theta_{error}^{install}=\theta (\rho^{\mathrm{detected}})-\theta (\rho^{in}).$$

Substituting Equation (20) into Equation (3), we find that the detected φ is:

(23) $$\varphi^{\mathrm{detected}}=\frac{1}{2}arc\tan(\frac{\sin2(\varphi +∆\alpha^{45})+\sin2(\varphi +∆\alpha^{135})}{\cos2(\varphi +∆\alpha^{0})+\cos2(\varphi +∆\alpha^{90})}).$$

Therefore, the azimuth error is:

(24) $$\;\psi_{errror}^{install}=\varphi^{in}-\varphi^{\mathrm{detected}}.$$

For the rotating polarizer imaging system, the polarizer is rotated at a specific angle by a turntable. The repeatability of the turntable is very high. Thus, the installation error is mainly the assembly error between the polarizer and the turntable. We can refer to Figure 4 to understand the installation error. This installation error is a fixed value, which is assumed to be:

(25) $$\;∆\alpha^{0}=∆\alpha^{45}=∆\alpha^{90}=∆\alpha^{135}=∆\alpha^{}.$$

Substituting Equation (25) into Equations (21) and (23), we have:

(26) $$\;\rho^{\mathrm{detected}}=\rho^{in}.$$

(27) $$\;\varphi^{\mathrm{detected}}=∆\alpha +\varphi^{in}.$$

Therefore, the azimuth error is:

(28) $$\;\psi_{errror}^{install}=∆\alpha .$$

That is to say, for rotating polarizer imaging, the installation error only affects the azimuth angle but not the zenith angle. The azimuth error is equal to the installation error of the polarizer.

### 3.3. Influence of the Different Noise on Polarization 3D Reconstruction Accuracy

In Section 3.1 and Section 3.2, we have deduced the influence of ER and installation error on polarization 3D imaging. Through the simulation of Section 4.1 and Section 4.2, we will find that as long as these two quantities are controlled within a certain range, the accuracy of the normal vector is hardly affected by them. On this basis, this section will continue to analyze the influence of noise on the accuracy of polarization 3D imaging. 

According to reference [37,38], the most important contribution to temporal noise of the CCD polarization imaging sensors are photon shot noise, readout electronic noise, thermal, and dark current noise etc. Although dark current contributes to sensor noise, for the CCD polarization sensors we currently have, it contributes less than 1 e- noise at operating temperature (40 °C). Therefore, for simplicity, the dark current will be ignored in this analysis. For the same reason, thermal noise could also be effectively controlled when the cooling system is used. Under this condition, thermal noise is a relatively small value compared to other noises. For simplicity, dark current and thermal noise will be ignored in this analysis. This section only focuses on the analysis of the impact of shot noise and readout noise on polarization 3D imaging. These two noises are closely related to the charge capacity and the number of A2D bit depth. We will quantitatively analyze the influence of these two parameters on polarization 3D reconstruction in Section 3.3.1 and Section 3.3.2, respectively.

#### 3.3.1. Influence of the Full-Well Capacity on Polarization 3D Reconstruction Accuracy

Polarization imaging has the characteristics of strong light weakening, so it is very easy to be disturbed by shot noise. For a single pixel in the CCD array, the number of photons collected during an integrating period is described by the Poisson distribution. We describe shot noise with the standard deviation of this Poisson distribution:

(29) $$\;\sigma_{shot}=\sqrt{\bar{I_{i}}}.$$

where $\bar{I_{i}}$ is the mean number of photons. The shot noise is independent and randomly affected by the number of photons per unit time.

Then, the noise of the polarization detector can be described by the following Equation:

(30) $$\;\sigma_{i}=\sqrt{\bar{e_{i}}+G^{2}}.$$

where $\bar{e_{i}}$ is the mean signal electrons of the polarized pixel whose rotation angle is *i* and *G* is the number of signal electrons equivalent to Gaussian noise. For typical polarization detection modes of 0, 45, 90, and 135°, the equivalent noise of each polarization direction can be expressed as:

(31) $$\begin{array}{l} \sigma_{0}=\sqrt{\bar{e_{0}}+G^{2}}\\ \sigma_{45}=\sqrt{\bar{e_{45}}+G^{2}}\\ \sigma_{90}=\sqrt{\bar{e_{90}}+G^{2}}\\ \sigma_{135}=\sqrt{\bar{e_{135}}+G^{2}} \end{array}.$$

According to the reference [37,38,42], we can use the error propagation principle to explore the influence of these noises on the zenith angle and azimuth angle. What we need to pay attention to is that error propagation formula is extremely dependent on the measurement scheme.

Commonly used polarization measurement schemes include (a) 0°, 45°, 90°; (b) 0°, 60°, 120°; and (c) 0, 45, 90, 135, etc. Scheme (a) and scheme (b) are the simplest schemes to solve the Stokes vector. However, scheme (c) has better structural symmetry, which is crucial for subsequent formula derivation. In addition, the signal-to-noise ratio (SNR) of the Stokes vector of scheme (c) is obviously better than scheme (a), and in certain cases, it will also be better than (b) too. Most importantly, we consider that the commercial DoFP polarization detectors such as Sony IMX250mzr and PolarCam both have adopted scheme (c), so we take scheme (c) as an example for analysis in this section.

The standard deviation of each Stokes vector component in scheme (c) is obtained as follows [38]:

(32) $$\begin{array}{l} \sigma_{s_{0}}=\sqrt{\bar{S_{0}}+2G^{2}}\\ \sigma_{s_{1}}=\sqrt{\bar{S_{0}}+2G^{2}}\\ \sigma_{s_{2}}=\sqrt{\bar{S_{0}}+2G^{2}} \end{array}.$$

In order to obtain the standard deviation of the ρ, we need to further transfer the Stokes vector standard deviation obtained above. According to the properties of Stokes vectors, *S*_0_, *S*_1_, and *S*_2_ are independent of each other such that the standard deviation of the ρ can be expressed by the following equation:

(33) $$\begin{array}{l} \sigma_{\rho }={\left.\sqrt{{\left(\frac{\partial \rho }{\partial S_{0}}\right)}^{2}\sigma_{S_{0}}^{2}+{\left(\frac{\partial \rho }{\partial S_{1}}\right)}^{2}\sigma_{S_{1}}^{2}+{\left(\frac{\partial \rho }{\partial S_{2}}\right)}^{2}\sigma_{S_{2}}^{2}}\right|}_{s_{0}={\bar{s}}_{0},s_{1}={\bar{s}}_{1},s_{2}={\bar{s}}_{2}}\\ \;\;\;=\sigma_{S_{0}}\sqrt{{\left(-\frac{\sqrt{{\bar{S}}_{1}^{2}+{\bar{S}}_{2}^{2}}}{{\bar{S}}_{0}^{2}}\right)}^{2}+{\left(\frac{{\bar{S}}_{1}}{{\bar{S}}_{0}\sqrt{{\bar{S}}_{1}^{2}+{\bar{S}}_{2}^{2}}}\right)}^{2}+{\left(\frac{{\bar{S}}_{2}}{{\bar{S}}_{0}\sqrt{{\bar{S}}_{1}^{2}+{\bar{S}}_{2}^{2}}}\right)}^{2}}\\ \;\;\;=\frac{\sigma_{S_{0}}}{{\bar{S}}_{0}}\sqrt{1+{\bar{\rho }}^{2}} \end{array}.$$

where $\overset{\_\_}{x}$ is the mean value of variable. In order to satisfy the linear mapping and improve the signal-to-noise ratio as much as possible, the optimal choice of $\bar{S_{0}}$ is $e_{well}$. Under this condition, Equation (33) can be rewritten as:

(34) $$\sigma_{\rho }==\frac{\sqrt{e_{well}+2\sigma_{guass}^{2}}}{e_{well}}\sqrt{1+{\bar{\rho }}^{2}}.$$

where $e_{well}$ is the full well capacity of the detector and $\sigma_{Gauss}$ is the Gaussian noise equivalent number of electrons. In order to simplify the derivation process, the Gaussian noise is considered equivalent to readout noise:$\sigma_{Gauss}=e_{well}/2^{N}$.

Then, Equation (34) becomes:

(35) $$\sigma_{\rho }==\sqrt{{\left(\frac{\sqrt{e_{well}}}{e_{well}}\right)}^{2}+2{\left(\frac{1}{2^{N}}\right)}^{2}}\sqrt{1+{\bar{\rho }}^{2}}.$$

If $e_{well}$ is small, N is big and $\frac{\sqrt{e_{well}}}{e_{well}}$ >> $\frac{1}{2^{N}}$. Equation (35) becomes:

(36) $$\sigma_{\rho }==\frac{\sqrt{e_{well}}}{e_{well}}\sqrt{1+{\bar{\rho }}^{2}}.$$

If the deviation is transmitted further, the standard deviation of the zenith angle can be obtained as:

(37) $$\sigma_{{}_{\theta }}^{full\_well}=\sqrt{{\left(\frac{\partial f}{\partial \rho }\right)}^{2}\sigma_{\rho }^{2}}.$$

where $\sigma_{{}_{\theta }}^{full\_well}$ is the standard deviation of the zenith angle caused by different full well capacity. Combining Equations (37) and (6), we can obtain $\sigma_{{}_{\theta }}^{full\_well}$. For simplicity, we define the following variables:

(38) $$\begin{array}{l} a=1+2\rho +\rho^{2}-2n^{2}+2n^{2}\rho +4n^{2}\rho^{2}-4n^{3}\rho \sqrt{\left(1-\rho )(\rho +1\right)}+n^{4}-n^{4}\rho^{2}\\ b=1+2\rho +\rho^{2}-2n^{2}+4n^{2}\rho +6n^{2}\rho^{2}+n^{4}+2n^{4}\rho +n^{4}\rho^{2} \end{array}.$$

Then, $\sigma_{{}_{\theta }}^{full\_well}$ is:

(39) $$\sigma_{{}_{\theta }}^{full\_well}=\frac{1}{2}\frac{1}{\sqrt{ab-a^{2}}}\frac{ab^{\prime }-a^{\prime }b}{b^{}}\sigma_{\rho }^{}.$$

We use the Monte Carlo [44] simulation to verify the above process. We set 5,000,000 random points that obey the Poisson distribution, which is consistent with the distribution of the photons. The results of the whole experiment are shown in Table 3.

In the same way, the standard deviation of the azimuth angle can be deduced as:

(40) $$\begin{array}{l} \sigma_{{}_{\psi \;}}^{full\_well}={\left.\sqrt{{\left(\frac{\partial \varphi }{\partial S_{1}}\right)}^{2}\sigma_{S_{1}}^{2}+{\left(\frac{\partial \varphi }{\partial S_{2}}\right)}^{2}\sigma_{S_{2}}^{2}}\right|}_{s_{0}={\bar{s}}_{0},s_{1}={\bar{s}}_{1},s_{2}={\bar{s}}_{2}}\\ \;\;\;=\sigma_{S_{0}}^{}\sqrt{{\left(-\frac{{\bar{S}}_{2}}{2({\bar{S}}_{1}^{2}+{\bar{S}}_{2}^{2})}\right)}^{2}+{\left(\frac{{\bar{S}}_{1}}{2({\bar{S}}_{1}^{2}+{\bar{S}}_{2}^{2})}\right)}^{2}}\\ \;\;\;=\frac{\sqrt{e_{well}}}{e_{well}}\frac{1}{2\bar{\rho }} \end{array}.$$

where $\sigma_{{}_{\psi \;}}^{full\_well}$ is the standard deviation of the azimuth angle caused by different full well capacity. The above error analysis can be used with the same method to conduct Monte Carlo experiments, so we will not go into details here.

#### 3.3.2. Influence of the A2D Bit Depth on Polarization 3D Reconstruction Accuracy

When the full-well size capacity is very large and A2D bit depth is small, the condition that $\frac{\sqrt{e_{well}}}{e_{well}}\gg \frac{1}{2^{N}}$ is not satisfied. In this case, the readout noise is relatively large, and Equation (39) becomes:

(41) $$\sigma_{{}_{\theta }}^{A2D}=\frac{1}{2}\frac{1}{\sqrt{ab-a^{2}}}\frac{a^{\prime }b-ab^{\prime }}{b^{}}\sqrt{{\left(\frac{\sqrt{e_{well}}}{e_{well}}\right)}^{2}+2{\left(\frac{1}{2^{N}}\right)}^{2}}\sqrt{1+{\bar{\rho }}^{2}}.$$

where $\sigma_{{}_{\theta }}^{A2D}$ is the standard deviation of the zenith angle caused by different A2D bit depths. In the same way, we can obtain the effect of A2D bit depths on the azimuth angle:

(42) $$\sigma_{{}_{\psi }}^{A2D}=\frac{1}{2\bar{\rho }}\sqrt{{\left(\frac{\sqrt{e_{well}}}{e_{well}}\right)}^{2}+2{\left(\frac{1}{2^{N}}\right)}^{2}}.$$

where $\sigma_{{}_{\psi }}^{A2D}$ is the standard deviation of the azimuth angle caused by differntA2D bit different depths.

## 4. Simulation and Analysis

From Section 3, we have established mathematical models for calculating zenith and azimuth angle errors. In this section, we will obtain the polarization 3D imaging error under various polarization detector performance parameters through simulation.

### 4.1. Simulation and Analysis of the Influence of ER on Polarization 3D Reconstruction

In Section 3.1, we derived the influence of the polarizer ER on polarization 3D imaging. We know that the ER influences the zenith angle but not the azimuth angle.

Referring to Equation (17), we set ER = 20, 40, 60, 80, 100, 200, and 400, and calculated the zenith angle error for each value.

**Figure 5:**
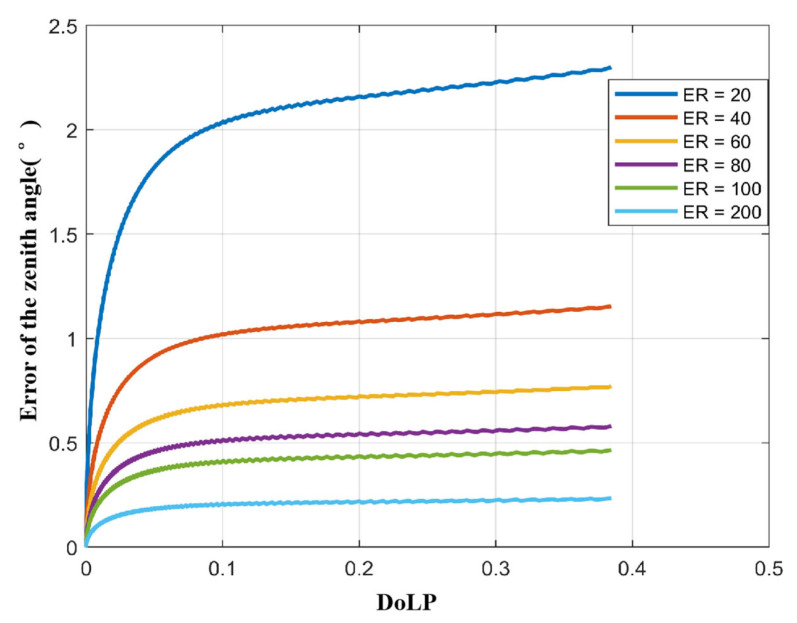
Influence of the extinction ratio (ER) on the zenith angle under the condition of different ρ of the light reflected from the target surface.

The results are shown in Figure 5: (a) The zenith angle error gradually decreases with the increase in ER; (b) For the same ER, the zenith angle error increases with increasing ρ. This means that the larger the zenith angle, the larger the zenith angle error caused by the ER.

Therefore, to improve polarization 3D imaging accuracy, it is necessary to improve the ER. However, we need to consider two things: (a) When ER = 200, the zenith angle error is already less than 0.25°, which is already a fairly good value in polarization 3D imaging experiments; (b) When ER > 200, the zenith angle accuracy improves very slowly. Considering the cost of the polarizer, it is uneconomical to continue to increase the extinction ratio.

Therefore, ER = 200 is our recommended value. However, users can also use the method provided in this paper to select a suitable ER according to their own requirements for 3D imaging accuracy.

**Figure 6:**
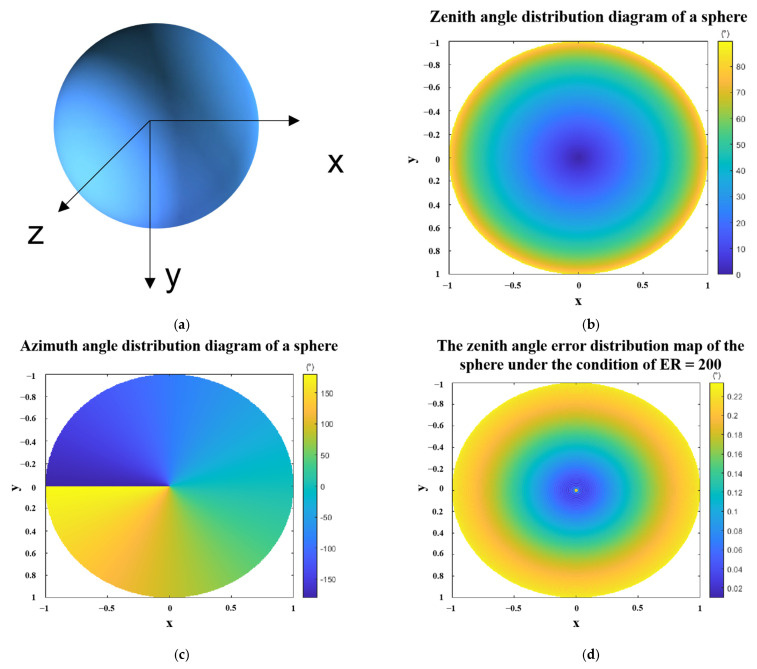
Schematic diagram of the zenith angle errors of a sphere under the condition of ER = 200. (**a**) schematic diagram of a sphere with a radius of 1. (**b**) zenith angle distribution of a sphere (**c**) azimuth angle distribution of a sphere. (**d**) zenith angle error distribution map of the sphere under the condition of ER = 200. Where x, y, z are the spatial coordinate axes, and the radius of the sphere is 1.

In order to show the zenith angle error of polarization 3D imaging more clearly, we have used the software to simulate the zenith angle error when ER is equal to 200. The result is shown in Figure 6. From the simulation results in Figure 6, the distribution of errors is consistent with our previous analysis. Only near the outer edge of the sphere, the error of the zenith angle is relatively large. Because the closer to the outside, the larger the zenith angle; that is, the larger ρ.

### 4.2. Simulation and Analysis of the Influence of Installation Error on Polarization 3D Reconstruction

In Section 3.2, we derived the influence of installation error on polarization 3D imaging accuracy. It can be seen from Equations (22) and (24) that the optimal choice is to maintain the same installation error in all four directions. However, this condition is generally only satisfied in a rotating polarizer imaging system.

For a rotating polarizer imaging system, the installation errors only affect the azimuth angle, which is a simple case that is not further analyzed here.

**Figure 7:**
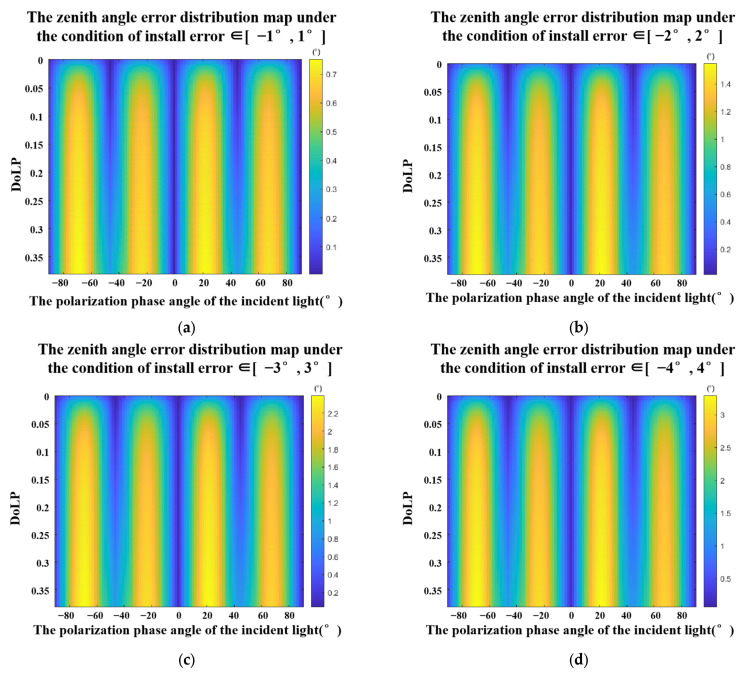
Zenith angle errors under the conditions of [Δα0, Δα45, Δα90, Δα135] ∈ (**a**). [−1°,1°], (**b**). [−2°,2°], (**c**). [−3°,3°], and (**d**). [−4°,4°].

For the DoFP detector, installation error may not be consistent in all four directions and the situation is more complicated. From Equation (22), it can be seen that the installation error is a factor that affects the accuracy of the zenith angle and the azimuth angle. For the convenience of analysis, we assume that the manufacturing tolerances are consistent in the four directions. Then, we use the Monte Carlo method to simulate and calculate the error of the zenith angle in different ranges. In our experiment, we set 4 ranges [−1°, 1°], [−2°, 2°], [−3°, 3°], and [−4°, 4°]. In each range, we set 1,000,000 sets of random arrays [Δα0, Δα45, Δα90, Δα135]. The result is shown in Figure 7. Through the simulation results, we found the following phenomena: (a) The zenith angle error decreases with the decrease in the installation error and (b) Under the same installation errors range, the zenith angle errors change more rapidly with the polarization phase angle of the incident light and has obvious periodicity.

**Figure 8:**
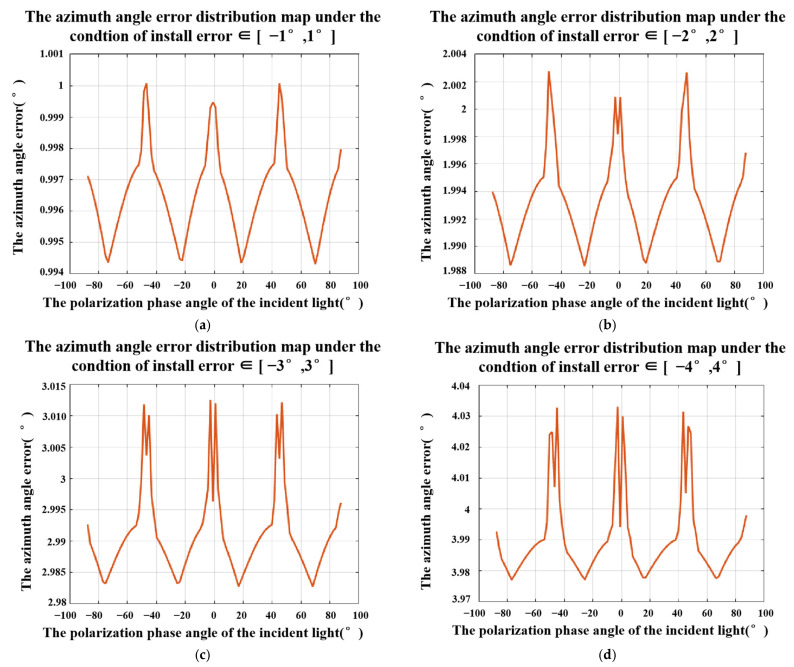
Azimuth angle errors under the conditions of [Δα0, Δα45, Δα90, Δα135] ∈ (**a**) [−1°,1°], (**b**) [−2°,2°], (**c**) [−3°,3°], and (**d**) [−4°,4°].

The same method can be used to simulate the azimuth errors caused by the installation error. The result is shown in Figure 8. We can get similar conclusions as above: (a) the error of the azimuth angle decreases with the decrease in the installation error and (b) Under the same installation errors range, the errors of the azimuth angle change with the change of the polarization phase angle of the incident light and has obvious periodicity.

**Figure 9:**
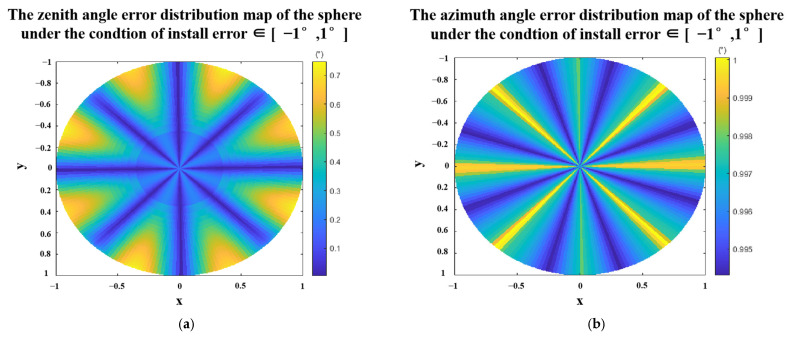
Polarization 3D imaging accuracy of the sphere with the install error ∈ [−1°,1°] (**a**) zenith angle errors distribution map. (**b**) azimuth angle errors distribution map. Where x, y, z are the spatial coordinate axes, and the radius of the sphere is 1.

Combining Figure 7 and Figure 8, we found that if the installation error is controlled within 1°, the zenith angle and azimuth angle errors can be well controlled. Similarly, we use a sphere to analyze the error distribution map of the zenith angle and the azimuth angle within 1° of the installation error. The results are shown in Figure 9. It can be seen from the error distribution maps that both the zenith angle error and the azimuth angle error have obvious periodicity with respect to the polarization phase angle of the incident light.

### 4.3. Simulation and Analysis of the Influence of Full-Well Size Capacity on Polarization 3D Reconstruction

In Section 3.3.1, we derived the influence of full well capacity on polarization 3D imaging accuracy. In this section, we will calculate these errors by setting different full well size capacity parameters. In the simulation experiment, we analyze the following common full well size capacity values: 10 *Ke*^−^, 100 *Ke*^−^, and 1 Me-.

**Figure 10:**
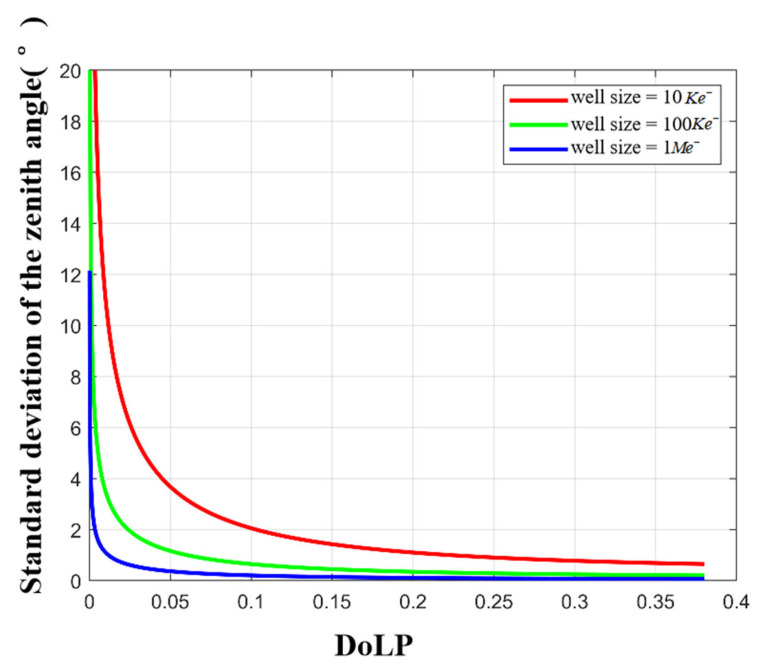
Zenith angle standard deviation for different full-well capacities.

**Figure 11:**
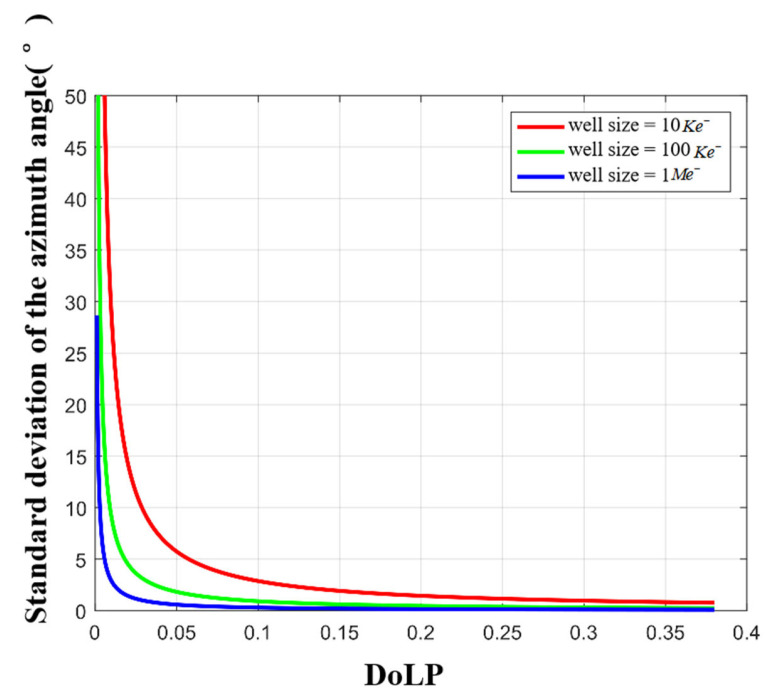
Azimuth angle standard deviation with different full-well capacities.

We first simulate the influence of different full-well capacities on the zenith angle. The zenith angle standard deviation under different full-well capacities is shown in Figure 10. Then, we simulate the influence of the full well capacity on the azimuth angle. The azimuth angle standard deviation is shown in Figure 11.

It can be seen from Figure 10 that (a) the larger the full well capacity, the smaller the standard deviation of the zenith angle; (b) there is a threshold, and when the full well capacity is greater than this threshold, the accuracy of the zenith angle will increase slowly; and (c) under the same full well condition, the smaller the ρ of the incident light (or the smaller the zenith angle), the larger the standard deviation of the zenith angle. Similar conclusions also exist in Figure 11. Based on the above analysis, considering the cost and imaging accuracy of a large capacity camera, we recommend 100 $Ke^{-}$ for polarization imaging.

**Figure 12:**
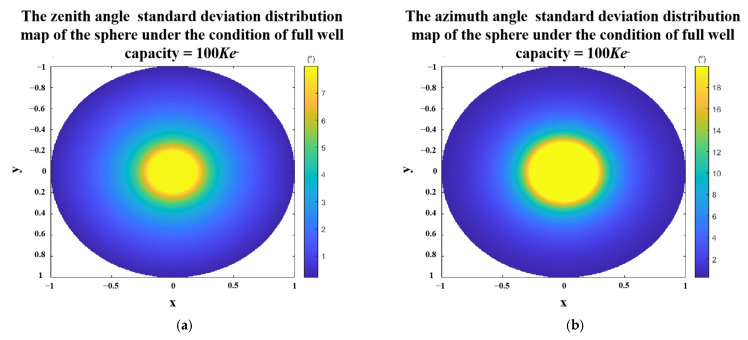
Polarization 3D imaging accuracy of the sphere with full well capacity equal to 100 *Ke*^−^. (**a**) standard deviation of zenith angle. (**b**) standard deviation of azimuth angle. Where x and y are the spatial coordinate axes, and the radius of the sphere is 1.

We still take the sphere as an example to simulate the standard deviation of the zenith angle and the azimuth angle under the condition of the full well capacity equals to 100 $Ke^{-}$ The results are shown in Figure 12. We found that the central part of the sphere, where the zenith angle is small, tends to have large standard deviations in both zenith and azimuth angles.

### 4.4. Simulation and Analysis of the A2D Bit Depth Influence on Polarization 3D Reconstruction

In Section 3.3.2, we analyzed the influence of A2D bit depth on polarization 3D imaging accuracy. In this section we calculate the standard deviations of the zenith and azimuth angles by simulation.

**Figure 13:**
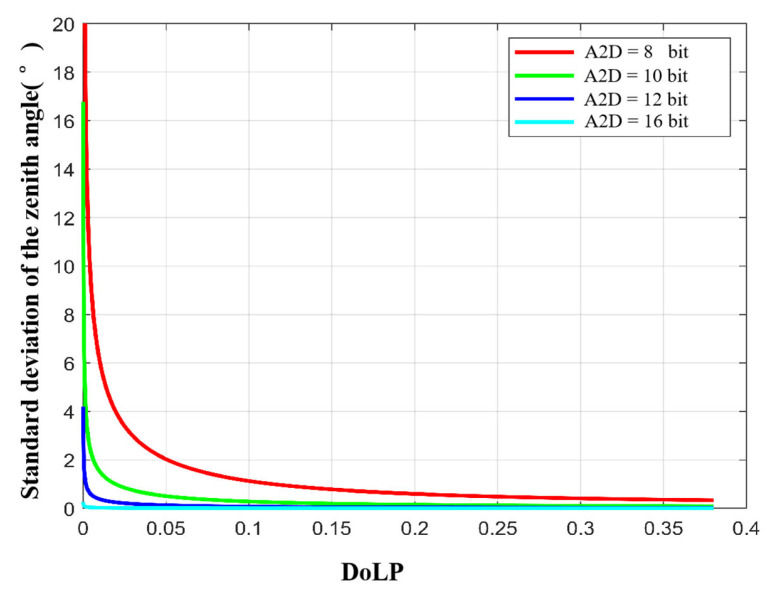
Standard deviation of the zenith angle with different A2D bit depths.

**Figure 14:**
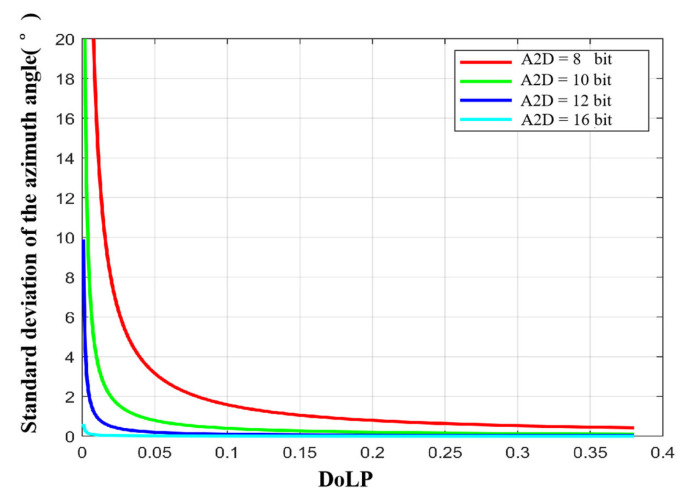
Standard deviation of the azimuth angle with different A2D bit depths.

To analyze the effect of A2D bit depth separately, we let the full well capacity approach infinity. Figure 13 and Figure 14 show the standard deviations of the zenith and azimuth angles at different A2D bit depths, respectively. We can see that (a) the standard deviations of zenith and azimuth angles decrease with increasing A2D bit depth; (b) there is a threshold, and when the A2D bit depth is greater than the threshold, increasing the A2D bit depth, the accuracy of the zenith angle and the azimuth angle increases slowly; (c) under the same A2D bit depth condition, the smaller the ρ of the incident light, the lager the standard deviation of the zenith angle and azimuth angle. Based on the above analysis and simulation results, we recommend using 12 bit for polarization 3D imaging.

**Figure 15:**
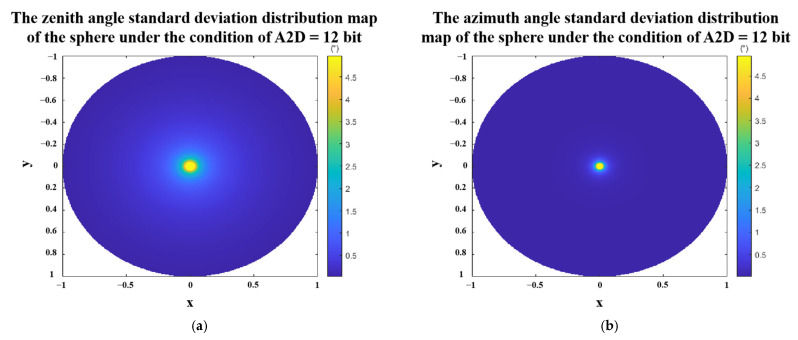
Polarization 3D imaging accuracy of the sphere with A2D bit depth equal to 12 bit. (**a**) standard deviation of zenith angle. (**b**) standard deviation of azimuth angle. Where x and y are the spatial coordinate axes, and the radius of the sphere is 1.

We still take the sphere as an example to simulate the standard deviation of the zenith angle and the azimuth angle under the condition that the A2D bit depth equals to 12. The results are shown in Figure 15. In the case of A2D bit depth equaling to 12 bit, only the zenith angle and azimuth angle errors in the middle of the sphere are large, and the errors in other places are small.

**Figure 16:**
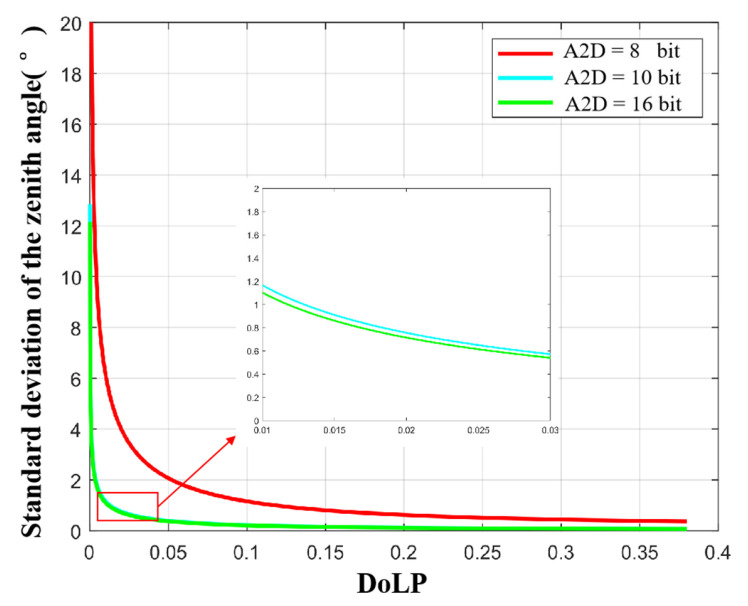
Standard deviation of the zenith angle at different A2D bit depths under the condition of the full well capacity equals to 1 Me-.

**Figure 17:**
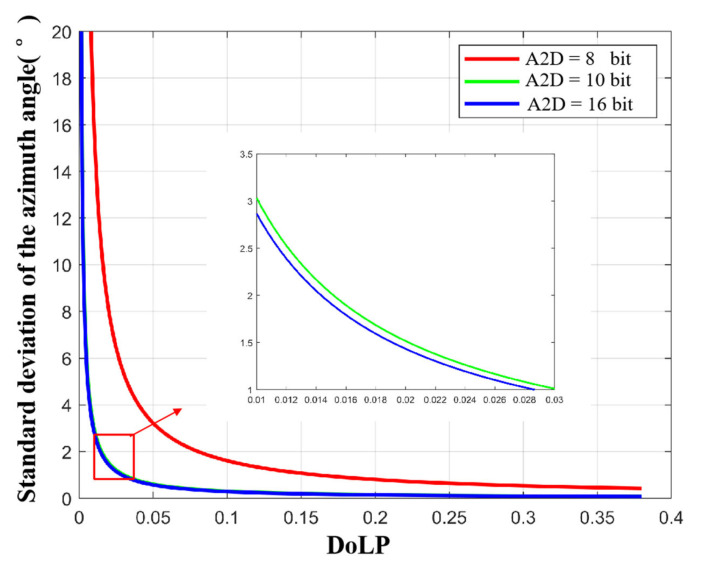
Standard deviation of azimuth angle at different A2D bit depths under the condition of full well capacity equals to 1 Me-.

Of course, it is impossible to realize that the full well capacity tends to infinity in the actual experiment process. Therefore, taking 1 Me- as a reasonable and existing large value of full well capacity, we analyzed the standard deviation of the zenith angle and the azimuth angle at different A2D bit depths to analyze the influence of A2D bit depth on polarization 3D imaging. The simulation results are shown in Figure 16 and Figure 17. At this time, the threshold in the above analysis is more obvious. When the A2D bit depth is greater than 12, the zenith angle and azimuth angle errors decrease very slowly.

## 5. Model Evaluation and Experiment

In Section 4, we simulated, and analyzed the zenith and azimuth angle errors for various parameters. In this section, we evaluate the model’s accuracy through experiments. While we can easily select various parameters during the simulation, only some representative parameters can be chosen experimentally to verify whether the error model established in this paper is consistent with the real error.

### 5.1. Establishment of the Experimental Platform

**Figure 18:**
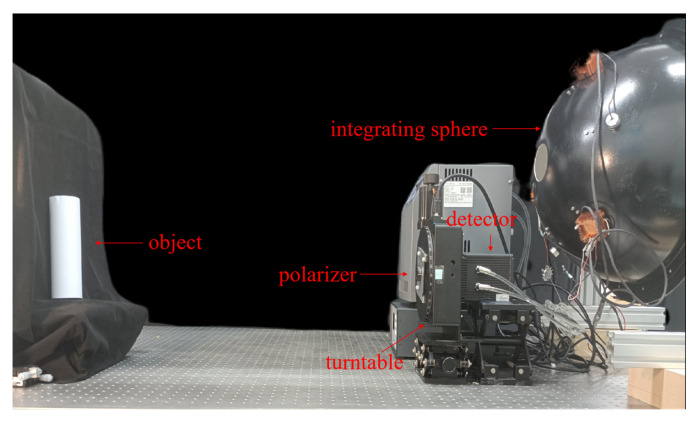
Photograph of the actual polarization experiment platform.

**Figure 19:**
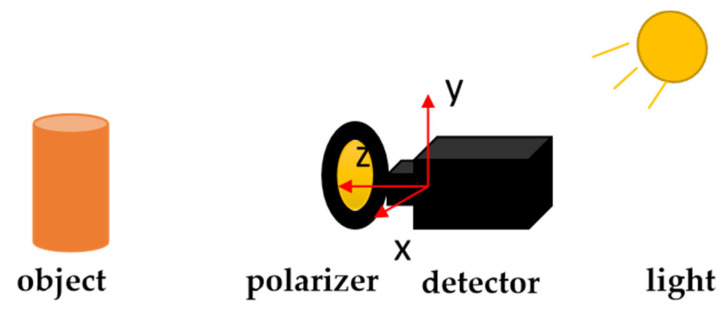
Schematic diagram of the experimental platform.

We chose the “detector + polarizer” method to conduct our experiment. The experimental platform was built as shown in Figure 18 and Figure 19. During the experiment, a high-precision turntable was used to drive the polarizer and form the polarization imaging system. The camera uses a dhyana v2 95 produced by China Xintu Photoelectric Co., Ltd., and its full-well charge number is 100 *Ke*^−^. The experimentally imaged object was a cylindrical paper cup. The experiment was conducted in a dark room. The specific experimental configuration is shown in Table 4.

### 5.2. Evaluation of Error Model Accuracy 

#### 5.2.1. Evaluation of the Effect of the Polarizer Extinction Ratio (ER) on the Polarization 3D Imaging Model

**Figure 20:**
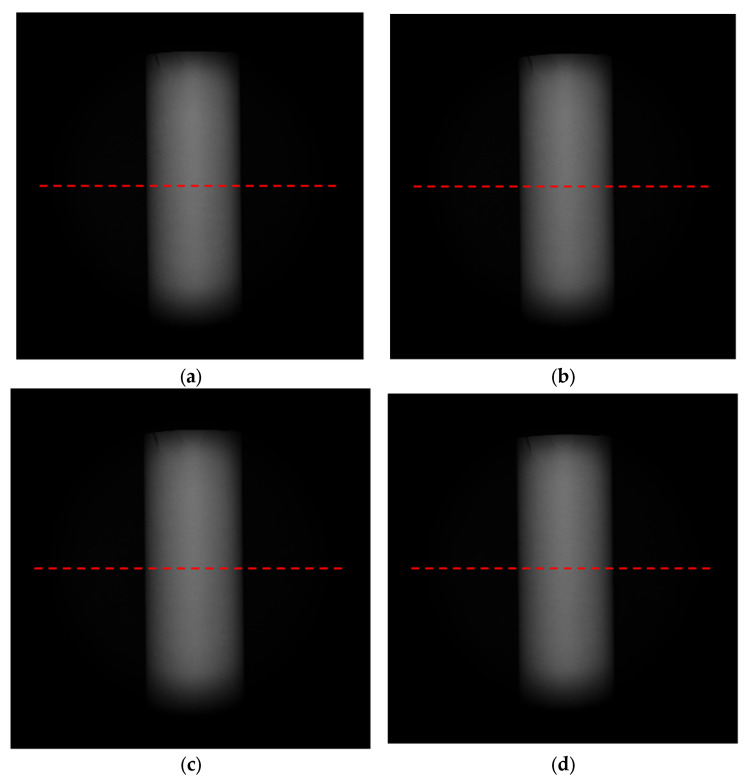
Experimentally collected polarization 3D images at (**a**) 0°, (**b**) 45°, (**c**) 90°, and (**d**) 135°.

In this section, a GCL-050004 polarizer produced by China Daheng Optoelectronics Co., LTD was used with an ER of 100. We used a high-precision turntable to drive the polarizer to rotate 0, 45, 90, and 135° to obtain polarization images. We captured 500 raw images with 16 bits in four directions and took the average value to reduce noise interference. The result after averaging in all directions is shown in Figure 20.

We selected row 1024 for analysis. In theory, the zenith angle distribution of row 1024 is [0°, 90°], but during the actual detection process, the zenith angle for most objects is less than 80°. Meanwhile, although we tried our best to suppress noise, some residual noise remained and greatly interfered when the zenith angle was less than 10°. In order to analyze the error due to ER, we only evaluated the accuracy of the error model under the condition of the zenith angle ∈ [10°, 80°]. 

We have to compare the actual (experimental) error and the theoretical error in the experiment. Actual (experimental) error is computed by using Equations (43) and (44):

(43) $$\theta \_{\mathrm{error}}_{actual}=abs(\theta_{}^{solved}-\theta^{theoretical}).$$

(44) $$\psi \_error_{actual}=abs(\psi_{}^{solved}-\psi^{theoretical}).$$

where $\theta_{}^{solved}$ is the solved zenith angle, $\theta^{theoretical}$ is the theoretical zenith angle, $\psi_{}^{solved}$ is the solved azimuth angle and $\psi^{theoretical}$ is the theoretical azimuth angle.

**Figure 21:**
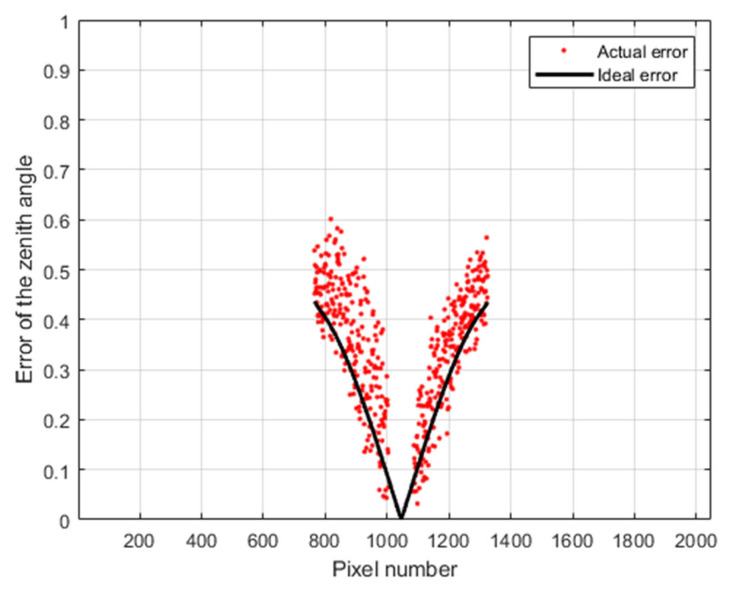
Actual (experimental) and theoretical zenith-angle error caused by the ER. Here pixel number is column number.

The experimental results are shown in Figure 21. We can see that the actual zenith-angle error is consistent with the error given by Equation (17).

Theoretically, the azimuth of row 1204 is 0° and this angle is not affected by the ER. However, we still observed error in the experiment, but it was caused by residual noise, not the ER.

#### 5.2.2. Evaluation of the Effect of Installation Error on the Polarization 3D Imaging Model

In the real world, installation error exists in the DoFP detector due to technological limitations. However, this error is difficult to measure. As an alternative, we used a turntable to simulate various errors. In our experiment, errors of 5, 10, 15, and 20° were artificially introduced in the four directions including 0°, 45°, 90°, and 135°. In order to reduce the influence of the ER, a WP25M-VIS polarizer produced by Thorlabs was selected along with the other experimental equipment described above.

**Figure 22:**
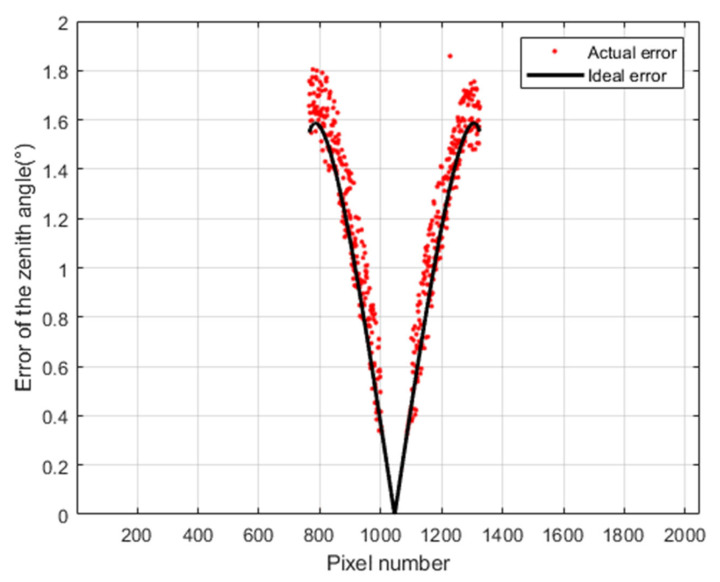
Actual (experimental) and theoretical zenith angle error caused by installation error. Here, pixel number is column number.

We still choose zenith angles ∈ [0°, 80°] to verify the accuracy of the error model. We compared the actual (experimental) error and the theoretical error. The actual (experimental) error is computed by Equations (43) and (44). The experiment results are shown in Figure 22. We can see that the actual zenith angle error is consistent with the error given by Equation (22).

Since the azimuth angle of row 1024 was zero, the theoretical azimuth angle error was 10.7752° under the experiment configuration. The average error of the row was calculated to be 11.8825°. That is to say, the theoretical azimuth angle error is consistent with the actual error. Based on the experiment results above, the error given by the model is consistent with the actual error.

#### 5.2.3. Evaluation of the Effect of Full-Well Capacity on the Polarization 3D Imaging Model

In Section 4.3, we simulated the effects of detector full-well capacity on polarization 3D imaging. In this section, we experimentally evaluate the accuracy of Equations (39) and (40). For this experiment, we changed the number of electrons in the detector by adjusting the exposure time. After adjusting the exposure time, 500 images of 12 bits of raw data were collected and zenith and azimuth angle values were calculated. We then calculated the standard deviation of the zenith and azimuth angles to verify model accuracy.

**Figure 23:**
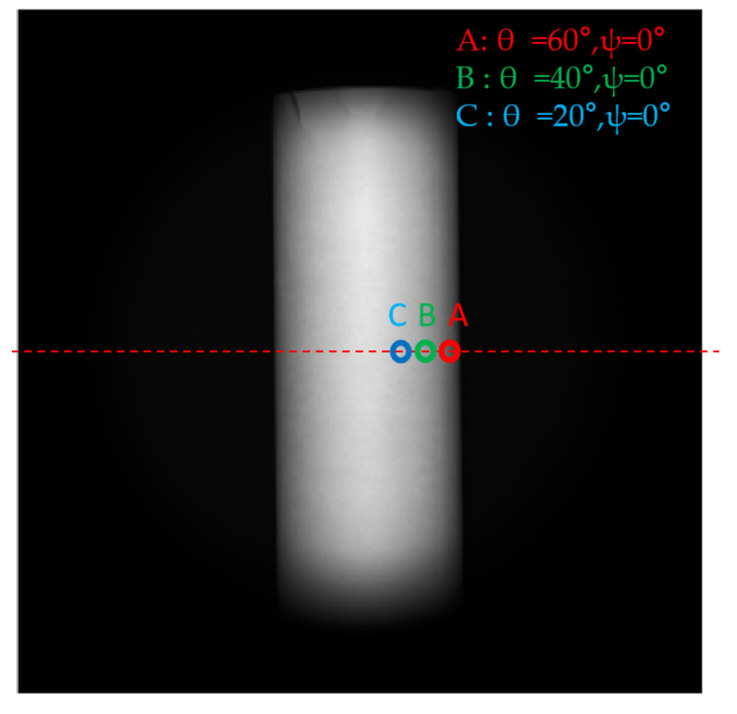
Typical points selected in the experiment for analysis.

In the case of a certain fixed exposure time, the number of electrons of each object point on the image is not consistent. As an alternative, we select several typical points to verify the accuracy of the model. In the experiment, points with zenith angles of 60, 40, and 20° in row 1024 were selected to verify whether the actual standard deviation of the zenith angle and azimuth angle were consistent with the standard deviation given by Equations (39) and (40). We refer to Figure 23 to understand these points. The equations for calculating the standard deviation of the actual zenith angle and azimuth angle are:

(45) $$\sigma_{\theta }^{actaul}=\sqrt{\frac{1}{500}\sum_{k=1}^{500}{\left(\theta_{k}^{solved}-\bar{\theta }\right)}^{2}}.$$

(46) $$\sigma_{{}_{\psi }}^{actual}=\sqrt{\frac{1}{500}\sum_{k=1}^{500}{\left(\psi_{k}^{solved}-\bar{\psi }\right)}^{2}}.$$

where $\theta_{k}^{solved}$ is the solved zenith angle of the *k*-th image, $\bar{\theta }$ is the theoretical zenith angle, $\psi_{k}^{solved}$ is the solved azimuth angle of *k*-th image, and $\bar{\psi }\;$ is the theoretical azimuth angle.

The experimental results are provided in Table 5 and Table 6. Based on the experimental results, the standard deviation given by the model is consistent with the actual standard deviation.

#### 5.2.4. Evaluation of the Effect of A2D Bit Depth on the Polarization 3D Imaging Model

In Section 4.4, we simulated the effects of detector A2D bit depth on polarization 3D imaging. In this section, we experimentally evaluate the accuracy of Equations (41) and (42).

It is difficult to evaluate the effect of A2D bit depth by ignoring the full well capacity of the detector during the actual experiment because we cannot achieve infinite full-well capacity. Therefore, the effect of full well capacity still exists in the experiment. In fact, Equations (41) and (42) also have parameters of full-well capacity. For specific effects of A2Dbit depth, please refer to Section 4.4. This section only evaluates the correctness of Equations (41) and (42). In the experiment, points with zenith angles of 60, 40, and 20° in row 1024 were selected to verify whether the actual standard deviation of the zenith angle and the azimuth angle was consistent with the standard deviation given by Equations (41) and (42). In this part of the experiment, Sony IMX250mzr sensor and dhyana v295 were used for the experiment, and non-uniformity correction and other operations were required before image collection. In the experiment, the intensity of the light source should be adjusted to keep the DN (digital number) of the measured point the same. The experimental results are provided in Table 7 and Table 8. Based on the experimental results, the error given by the model is consistent with the actual error.

## 6. Summary

This paper analyzed the relationship between the errors of polarization 3D imaging based on diffuse reflection and the performance parameters of the polarization detector. Several important factors affecting polarization 3D imaging accuracy were analyzed, including the polarizer ER, the polarizer installation error, the full well capacity, and the A2D bit depth. In order to explore the quantitative influences of these parameters on the polarization 3D imaging accuracy, the corresponding mathematical models were established. Through simulation analysis, suitable parameters were recommended, such as an ER ≥ 200, a full-well capacity ≥ 100 $Ke^{-}$, an installation error ≤ 1°, and an A2D bit depth ≥ 12 bit. Although we can further improve the above parameters to obtain better results, this would increase the manufacturing cost, and the accuracy of 3D reconstruction would be improved very slowly, which is not worth the loss. Finally, the accuracy of the theoretical error model was also evaluated by experiments.

According to the error models of polarization 3D imaging proposed in this paper, the researchers can choose the appropriate polarization detector according to their accuracy requirements, to reduce the model errors caused by the instrument. The proposed error models provide important theoretical support for the selection of the polarization detector suitable for 3D imaging, which is extremely crucial for the polarization 3D imaging—especially for single imaging represented by airborne polarization remote sensing 3D imaging. At the same time, in the era of deep learning, the method described in this paper also provides guidance for the production of polarization 3D imaging dataset. Based on this dataset, different polarization 3D imaging methods can be evaluated under the same evaluation system in the future. This is also the focus of the authors’ future work.

## Figures and Tables

**Table 1:** The normal vector error of different algorithms for polarization 3D imaging, expressed in the form of Mean Absolute Error (MAE) [27].

Scene	Yunhao [27]	Smith [25]	Mahmoud [24]	Miyazaki [30]
Box	23.31°	31.00°	41.51°	45.47°
Dragon	22.55°	49.16°	70.72°	57.72°
Father Christmas	13.50°	39.68°	39.20°	41.50°
Flamingo	20.19°	36.05°	47.98°	45.58°
Horse	22.27°	55.87°	50.55°	51.34°
Vase	10.32°	36.88°	44.23°	43.47°
Whole set	18.52°	41.44°	49.03°	47.51°

**Table 2:** Zenith angle solved with typical ρ values.

ρ	Zenith Angle, *θ* (°)
0.100	60.8439
0.095	59.7993
0.010	23.5136
0.005	16.8986

**Table 3:** Monte Carlo experiment results.

Variable	Theoretical Variance	Monte Carlo Variance
e_0_	470,000	469,880
e_45_	551,961	552,000
e_90_	530,000	531,001
e_135_	448,038	447,607
S_0_	1,000,000	999,290
S_1_	1,000,000	999,690
S_2_	1,039,200	1,000,500
ρ	0.000001014	0.000001007
*θ(°)*	0.000532430	0.000525900

**Table 4:** Equipment parameters in the experiments.

Camera Parameters	name	dhyana v2
	resolution	2048 × 2048
	Imaging distance	0.9–2.8 m
Object	object size	Height: 212 mm Diameter: 62 mm
	distance from detector	1 m
	object type	similar to Lambertian
Beam	device name	integrating sphere
	The aperture of the integrating sphere	100 mm
	Internal dimensions	Diameter: 500 mm
	Internal material	PEFT
	reflectivity	>98% (400–650 nm) >95% (300–800 nm)

**Table 5:** Actual and theoretical standard deviation of the zenith angle at the typical points with different electrons.

Point ID	Actual θ (°)	Exposure Time (ms)	Electrons (*Ke*^−^)	$\sigma_{\theta }^{\mathrm{theoretical}}\;({}^{\circ})$	$\sigma_{\theta }^{\mathrm{actual}}\;({}^{\circ})$	Difference (°)
A	60	300	35	1.1395	1.4429	0.3034
		250	33	1.1736	1.5664	0.3928
		200	29	1.2519	1.6782	0.4263
B	40	300	68	1.9410	2.3342	0.3932
		250	57	2.1201	2.6128	0.4927
		200	49	2.2866	2.8533	0.5667
C	20	300	94	4.3827	4.9181	0.5354
		250	78	4.8113	5.5123	0.7010
		200	65	5.2706	6.0344	0.7638

**Table 6:** Actual and theoretical standard deviation of the azimuth angle at the typical points with different electrons.

Point ID	Actual ψ (°)	Exposure Time (ms)	Electrons (*Ke*^−^)	$\sigma_{\psi }^{\mathrm{theoretical}}\;({}^{\circ})$	$\sigma_{\psi }^{\mathrm{actual}}\;({}^{\circ})$	Difference (°)
A	0	300	35	1.5969	1.8324	0.2355
		250	33	1.6446	1.9821	0.3375
		200	29	1.7543	2.2242	0.4699
B	0	300	68	3.3393	4.0117	0.6724
		250	57	3.6473	4.1237	0.4764
		200	49	3.9339	4.3123	0.3784
C	0	300	94	13.1608	14.0091	0.8483
		250	78	14.4477	14.9986	0.5509
		200	65	15.8268	16.7839	0.9571

**Table 7:** Actual and theoretical standard deviation of the zenith angle at the typical point with different A2D bit depth and electrons.

Point ID	Actual θ (°)	Electrons (*Ke*^−^)	A2D bit Depth	$\sigma_{\theta }^{\mathrm{theoretical}}\;({}^{\circ})\;$	$\sigma_{\theta }^{\mathrm{actual}}\;({}^{\circ})\;$	Difference (°)
A	60	9.8	8	2.4660	2.9123	0.4463
			12	2.1547	2.4134	0.2587
B	40	9.8	8	5.8550	6.2113	0.3563
			12	5.1159	5.3178	0.2019
C	20	9.8	8	15.5437	16.009	0.4653
			12	13.5815	13.904	0.3225

**Table 8:** Actual and theoretical standard deviation of the azimuth angle at the typical points different A2D bit depth and electrons.

Point ID	Actual ψ (°)	Electrons (*Ke*^−^)	A2D%bit Depth (bit)	$\sigma_{\psi }^{\mathrm{theoretical}}\;({}^{\circ})$	$\sigma_{\psi }^{\mathrm{actual}}\;({}^{\circ})$	Difference (°)
A	0	9.8	8	3.4556	3.9174	0.4618
			12	3.0194	3.5417	0.5223
B	0	9.8	8	10.0728	11.1219	1.0491
			12	8.8013	9.5416	0.7403
C	0	9.8	8	46.6756	48.1217	1.4461
			12	40.7836	42.1214	1.3378
		98	12	12.9672	13.4159	0.4487

## Data Availability

The data are contained within the article.
